# Integrated bioinformatics analysis reveals convergent molecular signatures associated with SERPINB7 and SERPINA12 deficiency

**DOI:** 10.3389/fmed.2026.1920631

**Published:** 2026-08-20

**Authors:** Zhenzhen Xiao, Yue Kang, Zixiao Zhao, Yue Zeng, Yunqian Zhuo, Fei Wang, Rui Li, Yingjian Tan

**Affiliations:** 1 Department of Dermatology, Fuzhou First General Hospital, Fuzhou, China; 2 Department of Respiratory and Critical Care, Xinxiang Central Hospital, Xinxiang, Henan, China; 3 Clinical Research Center for Hair and Skin Science, Department of Dermatology and Allergy, Charité - Universitätsmedizin Berlin, Berlin, Germany; 4 Department of Pathology, Fujian Medical University Cancer Hospital, Fujian Cancer Hospital, Fuzhou, China; 5 Department of Dermatology, Beijing Pinsu Medical Aesthetic Clinic, Beijing, China; 6 Department of Dermatology, Venereology and Allergology, Charité - Universitätsmedizin Berlin, Berlin, Germany

**Keywords:** extracellular matrix, PPK, SERPINA12, SERPINB7, skin inflammation

## Abstract

**Background:**

Pathogenic variants in *SERPINA12* have recently been identified in patients with palmoplantar keratoderma (PPK). Clinically, SERPINA12-associated PPK shares substantial overlap with Nagashima-type palmoplantar keratoderma (NPPK) caused by SERPINB7 deficiency, although inflammatory manifestations appear to be more prominent in SERPINA12-associated disease. The molecular basis underlying these similarities and differences remains unclear.

**Methods:**

Virtual knockout analyses of *SERPINA12* and *SERPINB7* were performed using the scTenifoldKnk framework. Functional enrichment analyses, including GO, KEGG, and GSEA, were conducted to characterize perturbed biological pathways. Correlation analyses were performed using extracellular matrix (ECM)- and inflammation-related gene signatures. In addition, *SERPINA12* was overexpressed in TNF-α-stimulated HaCaT keratinocytes to evaluate its effects on inflammatory cytokine expression.

**Results:**

Both *SERPINA12* and *SERPINB7* were associated with ECM-related biological processes and pathways, suggesting a potential shared role in extracellular matrix homeostasis. However, *SERPINA12* deficiency preferentially perturbed immune- and inflammation-related pathways, including Toll-like receptor signaling, interleukin signaling, and leukocyte activation. Consistently, *SERPINA12* overexpression significantly reduced TNF-α, IL-8, and IL-1β expression in TNF-α-stimulated HaCaT cells.

**Conclusion:**

*SERPINA12* and *SERPINB7* are associated with extracellular matrix-associated functions that may account for their similar keratoderma phenotypes. In contrast, *SERPINA12* additionally may exerts anti-inflammatory effects, providing a potential molecular explanation for the enhanced inflammatory manifestations observed in SERPINA12-associated PPK.

## 1 Introduction

Palmoplantar keratoderma (PPK) comprises a heterogeneous group of inherited disorders characterized by abnormal hyperkeratosis of the palms and soles (1–3). Depending on the underlying genetic defects, PPK can present as an isolated skin disease or as part of a syndromic disorder involving multiple organ systems (4–6). To date, pathogenic variants in numerous genes involved in epidermal differentiation, keratinization, cell adhesion, and protease regulation have been identified as causes of hereditary PPK (7). Despite considerable genetic heterogeneity, many forms of PPK share overlapping clinical features, suggesting the existence of common pathogenic mechanisms that remain incompletely understood (8, 9).

Nagashima-type palmoplantar keratoderma (NPPK) is one of the most common forms of autosomal recessive PPK, particularly in East Asian populations (10). NPPK is caused by loss-of-function variants in *SERPINB7*, a member of the serine protease inhibitor (SERPIN) superfamily (11, 12). Clinically, NPPK is characterized by diffuse erythematous hyperkeratosis of the palms and soles, excessive sweating, and extension of lesions beyond the palmoplantar surfaces (13). Previous studies have suggested that *SERPINB7* plays a protective role in maintaining epidermal integrity by regulating protease activity and preventing excessive tissue damage (14). Although several downstream molecular alterations associated with SERPINB7 deficiency have been reported (14–16), the comprehensive regulatory networks and molecular mechanisms underlying SERPINB7-associated palmoplantar keratoderma remain incompletely understood.

Members of the SERPIN superfamily participate in diverse biological processes, including protease inhibition, tissue remodeling, extracellular matrix (ECM) maintenance, and immune regulation (4). Growing evidence indicates that disruptions in ECM homeostasis contribute to epidermal barrier dysfunction and hyperkeratosis in various inherited skin diseases (14, 15). In parallel, inflammatory signaling pathways are increasingly recognized as important modifiers of disease severity and clinical heterogeneity in keratinization disorders (17, 18). However, whether *SERPINA12* and *SERPINB7* are associated with molecular mechanisms related to ECM regulation, and whether differential regulation of inflammatory pathways contributes to their distinct clinical phenotypes, remain unknown (18).

In the present study, we performed comparative transcriptomic analyses using virtual knockout models of *SERPINA12* and *SERPINB7*. Predicted perturbed genes and enriched biological pathways were identified and compared between the two models. Furthermore, correlation analyses focusing on inflammation-related and ECM-related genes were conducted to characterize the molecular networks associated with each gene. Our findings show that both *SERPINA12* and *SERPINB7* are closely associated with extracellular matrix regulation, supporting a potential shared pathogenic basis for their overlapping keratoderma phenotypes. In contrast, *SERPINA12* exhibits a stronger association with inflammatory signaling pathways, providing a potential molecular explanation for the enhanced inflammatory manifestations observed in SERPINA12-associated palmoplantar keratoderma (18).

## 2 Materials and methods

### 2.1 Single-cell transcriptomic dataset

A publicly available single-cell RNA sequencing dataset (GSE130973) was obtained from the Gene Expression Omnibus (GEO) database and used as the reference transcriptomic dataset for virtual knockout analysis (Supplementary Figure S1). The processed Seurat object generated by the original study was used for downstream analysis, and quality control, normalization, batch correction, and cell annotation were performed as described in the original publication. The dataset comprised multiple skin-resident cell populations, including keratinocytes, fibroblasts, endothelial cells, melanocytes, and immune cells. Virtual knockout analysis was performed using the scTenifoldKnk algorithm to predict regulatory perturbations associated with SERPINA12 or SERPINB7 deficiency. Genes predicted to be significantly perturbed were subsequently subjected to functional enrichment analyses, including Gene Ontology (GO), Kyoto Encyclopedia of Genes and Genomes (KEGG), Reactome, and MSigDB pathway analyses. Adjusted *p* values were calculated using the Benjamini–Hochberg method to control the false discovery rate.

### 2.2 Virtual knockout analysis using scTenifoldKnk

Virtual knockout analyses were performed using the scTenifoldKnk framework, a machine-learning–based computational approach for predicting the transcriptional consequences of gene deletion through gene regulatory network perturbation (19, 20). The quality-controlled and normalized Seurat object described above was used as input. Unless otherwise specified, analyses were performed using the default parameters recommended by the developers.

Briefly, scTenifoldKnk first reconstructed a wild-type gene regulatory network (GRN) from the scRNA-seq data using the scTenifoldNet workflow. During network construction, cells were randomly subsampled multiple times to generate independent cell subsets, and principal component regression was applied to each subset to infer directed and weighted gene regulatory networks. The resulting adjacency matrices were denoised using CANDECOMP/PARAFAC (CP) tensor decomposition and averaged to generate the final wild-type GRN.

To simulate gene loss-of-function, the wild-type adjacency matrix was duplicated, and the outgoing regulatory connections of the target gene (SERPINA12 or SERPINB7) were computationally removed by setting the corresponding row of the adjacency matrix to zero, thereby generating a pseudo-knockout GRN. The wild-type and pseudo-knockout GRNs were subsequently aligned using quasi-manifold alignment, and genes were projected into a shared low-dimensional latent space. Regulatory perturbation was quantified by calculating the Euclidean distance between the wild-type and pseudo-knockout representations of each gene. Genes were ranked according to the magnitude of perturbation, and significantly perturbed genes were identified using *χ*^2^ statistics with Benjamini–Hochberg false discovery rate (FDR) correction, with an adjusted *p* value < 0.05 considered statistically significant. Functional enrichment analysis was subsequently performed using the clusterProfiler package. Importantly, the log₂ fold change (log_2_FC) generated by scTenifoldKnk reflects the magnitude of predicted regulatory perturbation rather than conventional differential gene expression. The complete analytical workflow is illustrated in Supplementary Figure S1.

### 2.3 Identification of perturbed genes

Genes significantly influenced by *SERPINA12* or *SERPINB7* deletion were extracted based on the statistical criteria provided by the scTenifoldKnk workflow. The top perturbed genes were visualized using bar plots and volcano plots.

### 2.4 Gene ontology enrichment analysis

To investigate the biological functions associated with *SERPINA12* and *SERPINB7* deficiency, GO enrichment analyses were performed using an online bioinformatics platform.

Enrichment analyses were conducted in three GO categories: Biological Process (BP), Cellular Component (CC), Molecular Function (MF). GO terms with adjusted *p* values < 0.05 were considered significantly enriched.

### 2.5 KEGG pathway enrichment analysis

KEGG pathway enrichment analysis was performed using significantly perturbed genes identified by scTenifoldKnk (20). Pathways with adjusted *p* values < 0.05 were considered significantly enriched. Enrichment results were visualized using bubble plots.

### 2.6 Gene set enrichment analysis

To evaluate the global biological effects induced by *SERPINA12* and *SE*RPINB7 deficiency, Gene Set Enrichment Analysis (GSEA) was performed using genes ranked according to their perturbation *Z*-scores generated by scTenifoldKnk (20). Significantly enriched pathways were identified based on normalized enrichment scores (NES) and adjusted *p* values. Enrichment plots were generated to visualize representative pathways.

### 2.7 Correlation analysis of extracellular matrix-related genes

To assess the relationship between *SERPINA12*, *SERPINB7*, and ECM regulation, correlation analyses were performed between the target genes and a curated ECM-related gene set, including collagens, laminins, fibronectin, matrix metalloproteinases, and other ECM-associated genes. Correlation coefficients were calculated and visualized using heatmaps.

### 2.8 Correlation analysis of inflammation-related genes

To explore the potential involvement of *SERPINA12* and *SERPINB7* in inflammatory regulation, correlation analyses were performed using a predefined panel of inflammation-related genes, including cytokines, chemokines, and immune response mediators. The resulting correlation matrices were visualized using heatmaps to compare the inflammatory networks associated with *SERPINA12* and *SERPINB7*.

### 2.9 Statistical analysis

All statistical analyses were performed using the algorithms implemented in the scTenifoldKnk platform and the associated bioinformatics analysis tools. Unless otherwise specified, adjusted *p* values < 0.05 were considered statistically significant.

### 2.10 Cell culture

Human immortalized keratinocyte HaCaT cells were cultured in Dulbecco’s Modified Eagle Medium (DMEM; Gibco, USA) supplemented with 10% fetal bovine serum (FBS; Gibco, USA) and 1% penicillin–streptomycin at 37 °C in a humidified atmosphere containing 5% CO₂.

### 2.11 *SERPINA12* overexpression

HaCaT cells were transfected with a *SERPINA12* overexpression plasmid or the corresponding empty vector control using Lipofectamine 3,000 (Invitrogen, USA) according to the manufacturer’s instructions. Following transfection, cells were incubated for 48 h prior to subsequent experiments.

### 2.12 TNF-α stimulation

To establish an inflammatory keratinocyte model, transfected HaCaT cells were stimulated with recombinant human TNF-α (10 ng/mL) for 4 h.

### 2.13 Quantitative real-time PCR

Total RNA was extracted using TRIzol reagent (Invitrogen, USA) according to the manufacturer’s protocol. Complementary DNA (cDNA) was synthesized using a reverse transcription kit (Takara, Japan). Quantitative real-time PCR (qRT-PCR) was performed using SYBR Green Master Mix on a real-time PCR detection system. The relative expression levels of TNF-α, IL-8, IL-1β, and IL-6 were normalized to GAPDH and calculated using the 2^−ΔΔCt method. All experiments were performed in triplicate.

### 2.14 Statistical analysis of experimental data

Experimental data are presented as mean ± standard deviation (SD). Statistical analyses were performed using GraphPad Prism 9.0. Comparisons among multiple groups were performed using one-way analysis of variance (ANOVA) followed by Tukey’s multiple comparison test. A two-sided *p* value < 0.05 was considered statistically significant.

## 3 Results

### 3.1 Virtual knockout of *SERPINA12* and *SERPINB7* induces extensive transcriptional perturbations

**Figure 1:**
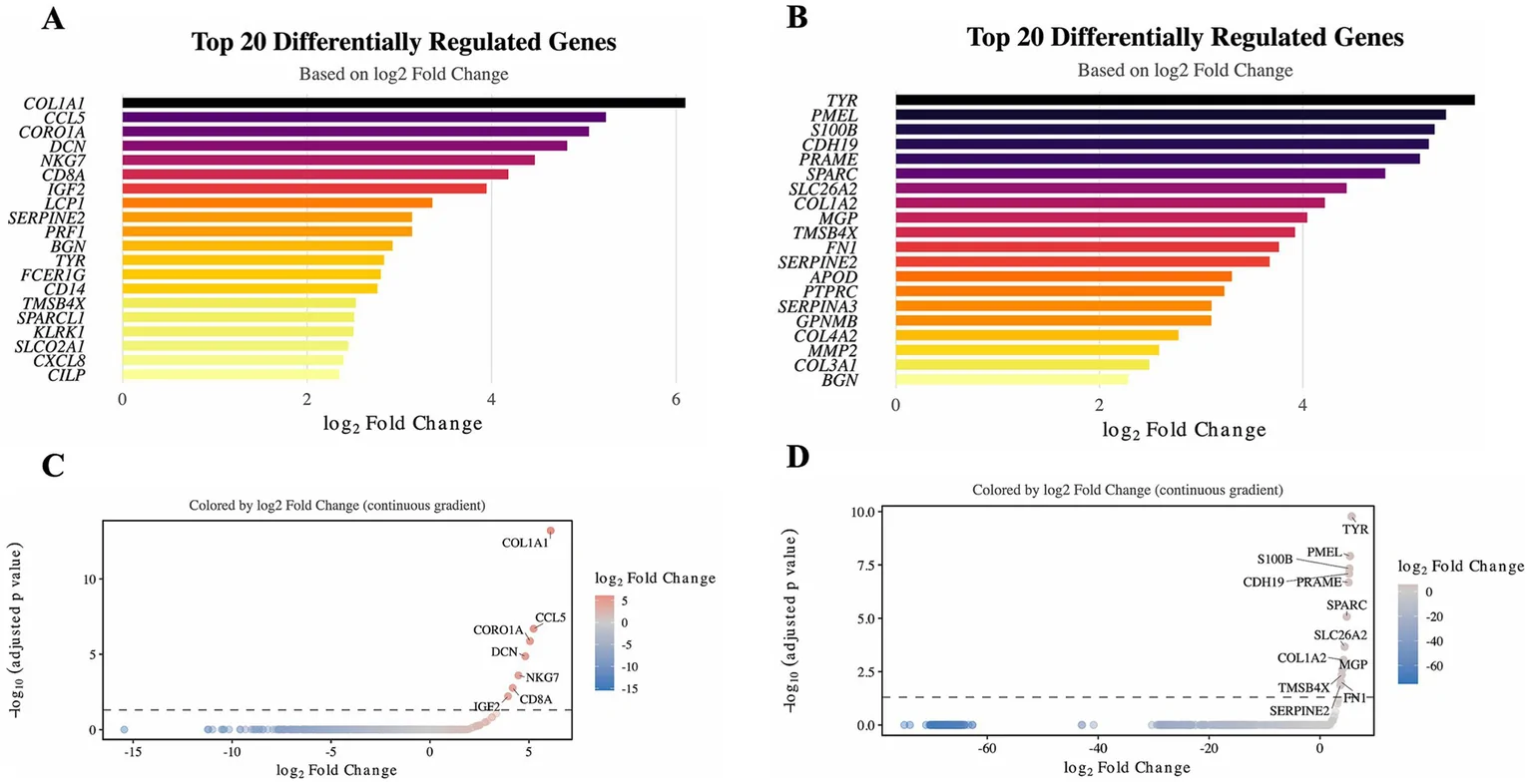
Comparative virtual knockout analysis identifies predicted perturbed genes associated with SERPINA12 and SERPINB7 deficiency. The top 20 predicted perturbed genes following virtual knockout of SERPINA12 **(A)** and SERPINB7 **(B)** are shown according to the predicted log_2_ fold change. Volcano plots illustrating predicted regulatory perturbations after virtual knockout of SERPINA12 **(C)** and SERPINB7 **(D)** are presented based on predicted log_2_ fold change and adjusted *p* values. Predicted perturbed genes with the greatest predicted perturbation are labeled.

To investigate the molecular consequences of *SERPINA12* and *SERPINB7* deficiency, virtual knockout analyses were performed using the scTenifoldKnk framework. Deletion of either gene resulted in substantial perturbations within the reconstructed gene regulatory networks (Figure 1). Following *SERPINA12* knockout, the most significantly perturbed genes included *COL1A1*, *CCL5*, *CORO1A*, *DCN*, *NKG7*, *CD8A*, *IGF2*, and *SERPINE2*. Notably, both extracellular matrix-related genes (*COL1A1* and *DCN*) and immune-associated genes (*CCL5*, *NKG7*, and *CD8A*) were among the top-ranked perturbed genes (Figures 1A,C), suggesting that *SERPINA12* may influence both tissue structural integrity and immune regulation.

In contrast, *SERPINB7* knockout predominantly affected genes involved in extracellular matrix organization and cell adhesion (Figures 1B,D), including *COL1A2*, *FN1*, *SPARC*, *MMP2*, and *COL4A2*. These findings suggest that *SERPINB7* deficiency primarily disrupts structural and extracellular matrix-associated networks. Collectively, these results suggest that both *SERPINA12* and *SERPINB7* contribute to the maintenance of epidermal homeostasis, although their downstream perturbation profiles exhibit distinct biological characteristics.

### 3.2 GO enrichment analysis provide evidence supporting a potential shared extracellular matrix-related functions but distinct downstream biological programs

**Figure 2:**
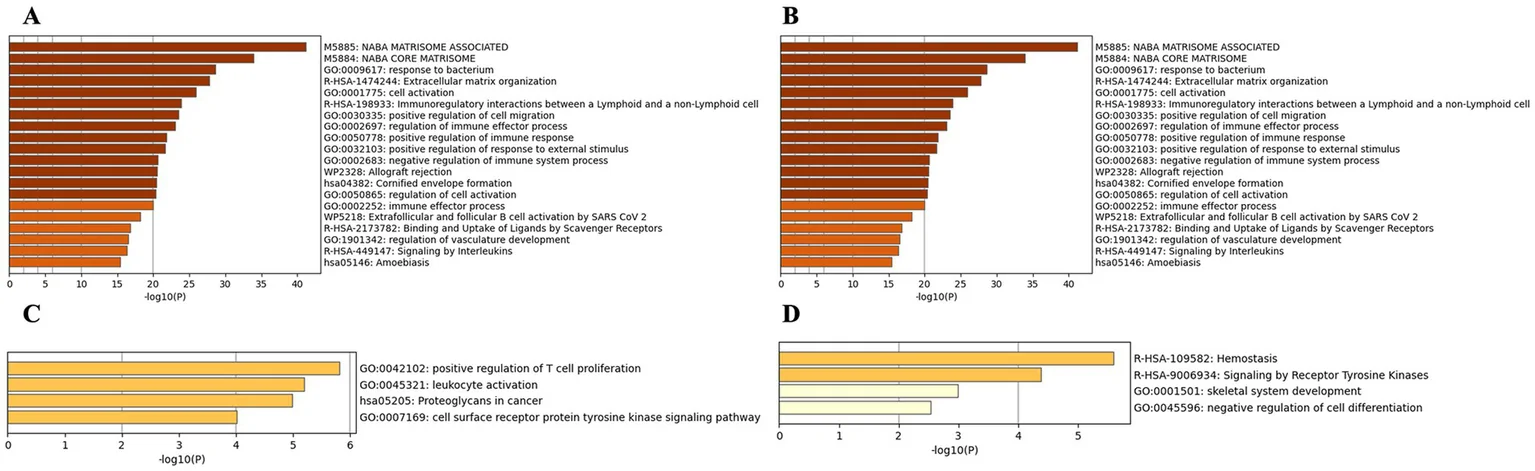
Functional enrichment analysis of genes perturbed by SERPINA12 and SERPINB7 virtual knockout. Functional enrichment analysis was performed using significantly perturbed genes predicted by the scTenifoldKnk algorithm. The top enriched biological pathways following virtual knockout of SERPINA12 **(A)** and SERPINB7 **(B)** are presented according to enrichment significance. Representative immune- and inflammation-related pathways associated with SERPINA12 deficiency **(C)** and pathways preferentially enriched following SERPINB7 deficiency **(D)** are also shown.

To further characterize the biological functions affected by *SERPINA12* and *SERPINB7* deficiency, Gene Ontology enrichment analyses were performed using genes perturbed following virtual knockout. Analysis of all perturbed genes demonstrated highly similar enrichment profiles between *SERPINA12* and *SERPINB7* (Figures 2A,B). The most significantly enriched terms included NABA Matrisome Associated, NABA Core Matrisome, extracellular matrix organization, and cell activation, indicating that both genes participate in extracellular matrix regulation and tissue homeostasis.

However, analysis of the most significantly perturbed genes revealed notable differences between the two knockout models (Figures 2C,D). SERPINA12-associated genes were enriched in immune-related processes, including positive regulation of T-cell proliferation, leukocyte activation, and cell surface receptor protein tyrosine kinase signaling pathways. In contrast, SERPINB7-associated genes were enriched in hemostasis, receptor tyrosine kinase signaling, skeletal system development, and negative regulation of cell differentiation.

These findings suggest that although *SERPINA12* and *SERPINB7* are associated with common extracellular matrix-associated functional framework, *SERPINA12* deficiency preferentially affects immune regulatory processes, whereas *SERPINB7* deficiency predominantly influences structural and differentiation-related pathways.

### 3.3 KEGG and GSEA analyses identify enhanced inflammatory pathway perturbation following *SERPINA12* deficiency

To compare the biological pathways affected by *SERPINA12* and *SERPINB7* deficiency, KEGG pathway enrichment and Gene Set Enrichment Analyses were performed. Both virtual knockout models exhibited significant enrichment of extracellular matrix-associated pathways. ECM–receptor interaction was identified as a common pathway affected by both *SERPINA12* and *SERPINB7* deletion, further supporting their shared role in extracellular matrix regulation.

**Figure 3:**
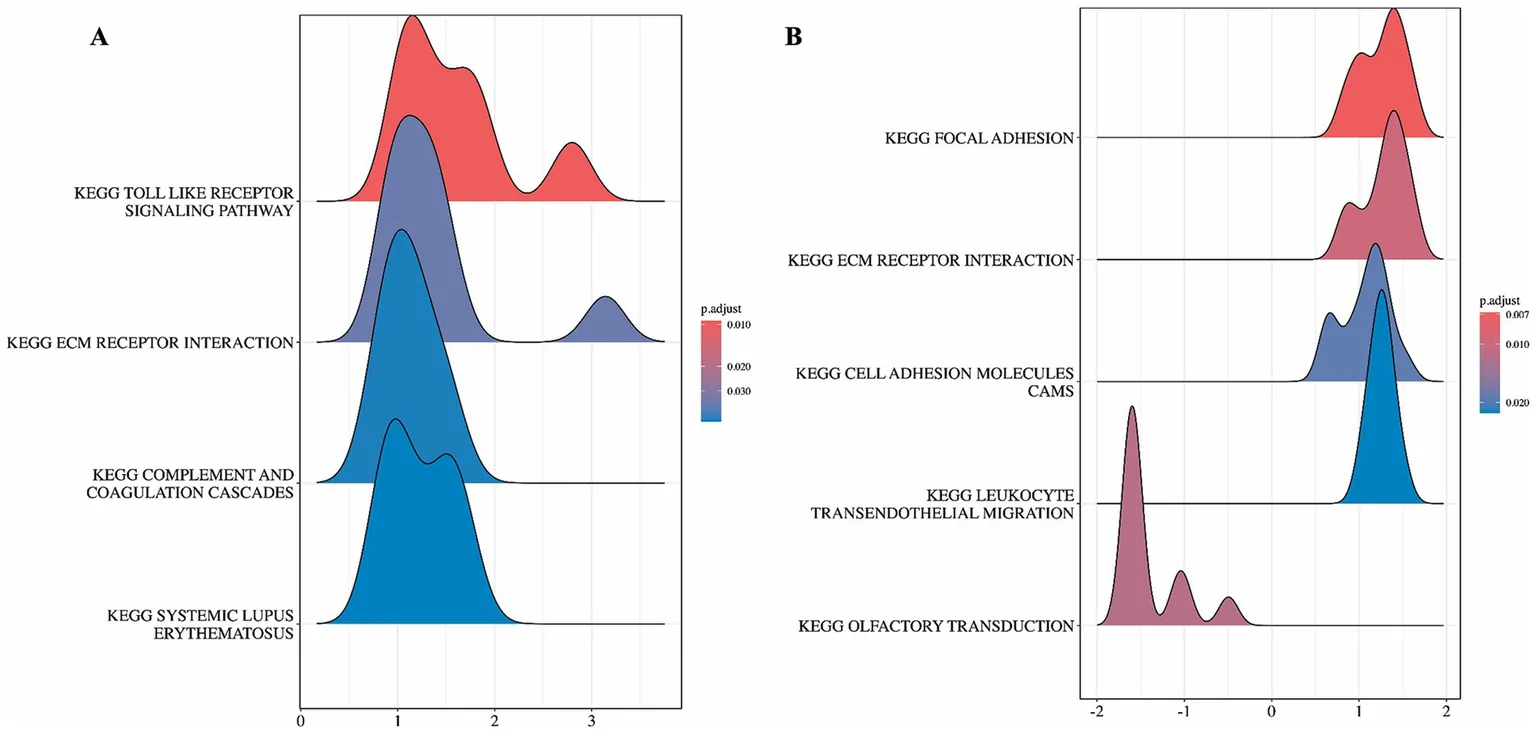
Gene set enrichment analysis of pathways associated with SERPINA12 and SERPINB7 deficiency. Gene set enrichment analysis (GSEA) was performed to identify biological pathways associated with virtual knockout of SERPINA12 **(A)** and SERPINB7 **(B)**. Representative enriched KEGG pathways are displayed as ridge plots, and the color gradient indicates the adjusted *p* value for each enriched pathway.

Notably, *SERPINA12* deficiency was additionally associated with several immune and inflammatory pathways, including Toll-like receptor signaling, complement and coagulation cascades, interleukin signaling, and immune response-related biological processes (Figure 3). GSEA analysis further demonstrated enrichment of immune-associated gene signatures following *SERPINA12* deletion. By contrast, *SERPINB7* deficiency was more strongly associated with focal adhesion, cell adhesion molecules, and structural remodeling pathways. Although certain immune-related pathways were also identified, their enrichment was less prominent than that observed following *SERPINA12* deletion.

Taken together, these results suggest that extracellular matrix dysregulation represents a potential shared molecular consequence of *SERPINA12* and *SERPINB7* deficiency, whereas enhanced immune and inflammatory perturbations may distinguish SERPINA12-associated disease.

### 3.4 Correlation analysis confirms associations with extracellular matrix and inflammatory networks

**Figure 4:**
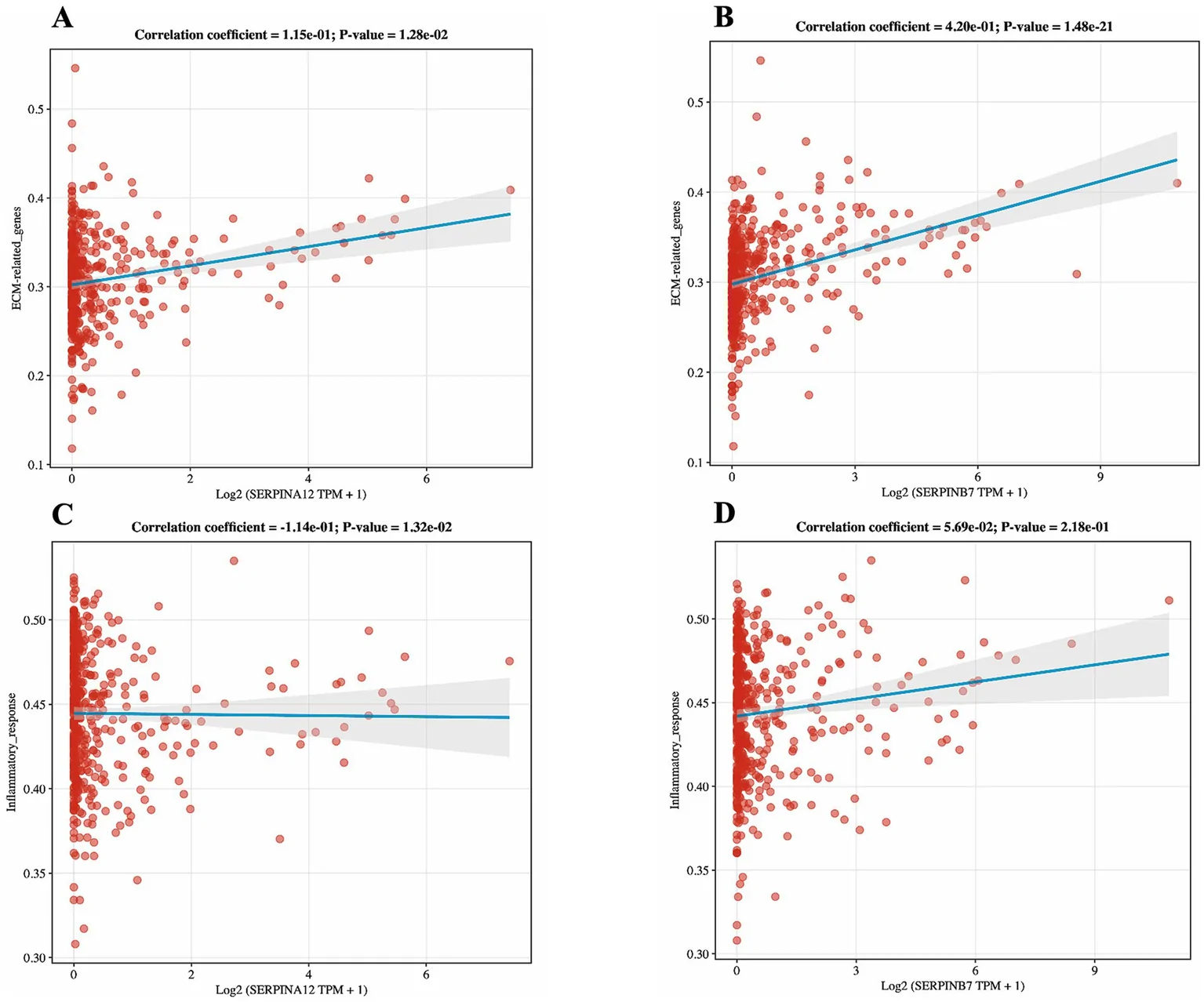
Correlation analysis between SERPINA12, SERPINB7, extracellular matrix-related genes, and inflammatory-response signatures. Correlation analyses were performed between SERPINA12 expression and extracellular matrix (ECM)-related gene signatures **(A)**, SERPINB7 expression and ECM-related gene signatures **(B)**, SERPINA12 expression and inflammatory-response signatures **(C)**, and SERPINB7 expression and inflammatory-response signatures **(D)**. Correlation coefficients and corresponding *p* values are indicated above each panel.

To further explore the functional relationships between *SERPINA12*, *SERPINB7*, and disease-related molecular pathways, correlation analyses were performed using extracellular matrix-related and inflammation-related gene signatures (Figure 4). Both *SERPINA12* and *SERPINB7* expression were significantly associated with extracellular matrix-related gene sets. However, the association was substantially stronger for *SERPINB7* (*r* = 0.42, *p* = 1.48 × 10^−21^) than for *SERPINA12* (*r* = 0.115, *p* = 0.0128), supporting a central role of *SERPINB7* in extracellular matrix homeostasis (Figures 4A,B).

Inflammation-related correlation analysis revealed a significant but weak association between *SERPINA12* expression and inflammatory response signatures (*r* = −0.114, *p* = 0.0132), whereas no significant association was observed for *SERPINB7* (*r* = 0.057, *p* = 0.218) (Figures 4C,D). Although the magnitude of the correlation was modest, these findings are consistent with the enrichment of immune- and inflammation-related pathways identified in the virtual knockout analyses. Overall, the correlation analyses support a model in which *SERPINB7* is more tightly linked to extracellular matrix regulation, whereas *SERPINA12* exhibits additional associations with inflammatory signaling networks.

### 3.5 *SERPINA12* overexpression attenuates TNF-α-induced inflammatory responses in keratinocytes

Although SERPINA12 is expressed in epidermal keratinocytes *in vivo*, endogenous SERPINA12 expression was negligible in HaCaT cells under our culture conditions. To experimentally validate the predicted involvement of *SERPINA12* in inflammatory regulation, *SERPINA12* overexpression plasmids were transfected into HaCaT keratinocytes followed by TNF-α stimulation. The expression levels of several inflammatory cytokines were subsequently assessed by quantitative PCR.

**Figure 5:**
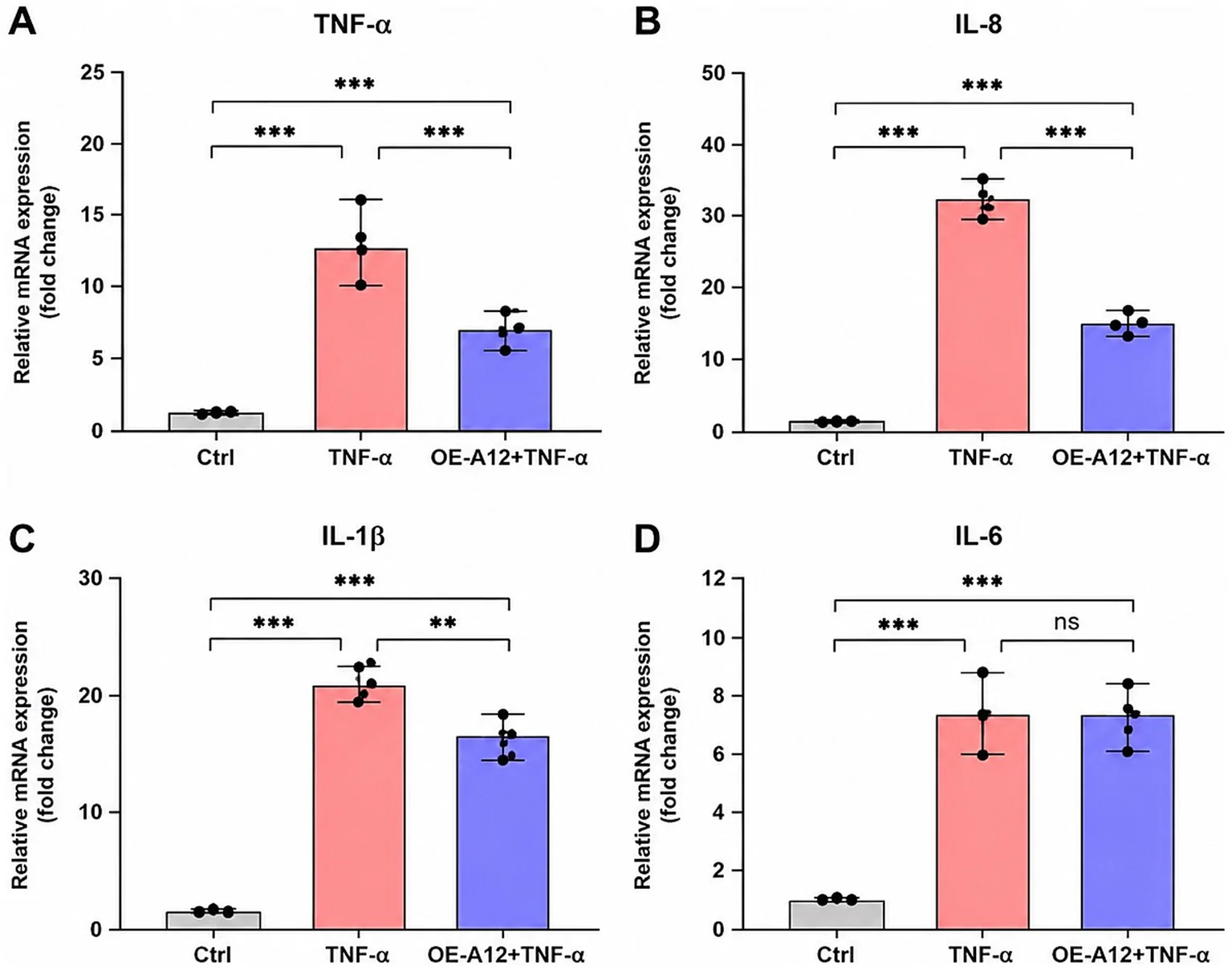
SERPINA12 overexpression attenuates TNF-α-induced inflammatory responses in HaCaT keratinocytes. HaCaT cells were transfected with a SERPINA12 overexpression plasmid (OE-A12) or the corresponding control vector before stimulation with TNF-α. Relative mRNA expression levels of TNF-α **(A)**, IL-8 **(B)**, IL-1β **(C)**, and IL-6 **(D)** were determined by quantitative real-time PCR (qRT-PCR). Data are presented as mean ± SD from three independent experiments. Statistical significance was determined using one-way ANOVA followed by multiple-comparison analysis. OE-A12 indicates SERPINA12 overexpression. *p* < 0.05 was considered statistically significant (*p* < 0.05, *p* < 0.01, *p* < 0.001; ns, not significant).

As expected, TNF-α stimulation markedly increased the expression of pro-inflammatory mediators compared with untreated control cells. Notably, *SERPINA12* overexpression significantly attenuated TNF-α-induced inflammatory responses (Figure 5). Relative TNF-α expression was reduced from 12.82 ± 0.19-fold in the TNF-α-treated group to 7.13 ± 0.26-fold in *SERPINA12*-overexpressing cells (*p* < 0.001). Similarly, IL-8 expression decreased from 34.09 ± 0.29-fold to 14.95 ± 0.38-fold following *SERPINA12* overexpression (*p* < 0.001). IL-1β expression was also significantly reduced from 21.36 ± 0.73-fold to 16.34 ± 0.40-fold (*p* < 0.01). In contrast, no significant difference was observed in IL-6 expression between the TNF-α-treated and SERPINA12-overexpressing groups (7.42 ± 0.11 vs. 7.74 ± 0.16, *p* > 0.05).

These findings indicates that *SERPINA12* suppresses the expression of multiple inflammatory mediators in keratinocytes and support the computational prediction that *SERPINA12* participates in the regulation of inflammatory signaling pathways. Together with the enrichment of Toll-like receptor signaling, interleukin signaling, and immune activation pathways identified in the virtual knockout analyses, these experimental results suggest that loss of *SERPINA12* may contribute to inflammatory responses in palmoplantar keratoderma.

## 4 Discussion

PPK comprises a heterogeneous group of inherited disorders characterized by excessive hyperkeratosis of the palms and soles (2, 21). Although numerous causative genes have been identified, the molecular mechanisms underlying the phenotypic similarities and differences among distinct forms of hereditary PPK remain incompletely understood (22). Recently, pathogenic variants in *SERPINA12* have been reported in patients presenting with diffuse palmoplantar keratoderma (12, 23). Clinically, SERPINA12-associated PPK exhibits remarkable overlap with NPPK, which is caused by loss-of-function variants in *SERPINB7* (18). Both disorders are characterized by diffuse palmoplantar hyperkeratosis and erythema, suggesting that these two members of the SERPIN family may participate in common biological pathways (22). Recent evidence has identified patients with palmoplantar keratoderma harboring heterozygous variants in both *SERPINB7* and *SERPINA12* (24, 25). The occurrence of disease in these individuals suggests that defects in multiple SERPIN family members may act synergistically to impair epidermal integrity and keratinization. These findings expand the genetic basis of palmoplantar keratoderma and support the concept of digenic inheritance in selected cases. However, clinical observations have suggested that inflammatory manifestations appear to be more pronounced in individuals carrying *SERPINA12* variants (18). The molecular basis underlying these similarities and differences has remained largely unknown.

In the present study, we performed a systematic comparison of *SERPINA12* and *SERPINB7* using a virtual knockout strategy based on the scTenifoldKnk framework. By integrating virtual knockout analyses with GO enrichment, KEGG pathway analysis, GSEA, gene-signature correlation analysis, and experimental validation in keratinocytes, we provide evidence for both shared and distinct molecular programs associated with these two genes. Our findings support a model in which extracellular matrix dysregulation represents a potential common pathogenic mechanism underlying both disorders, whereas inflammatory regulation distinguishes *SERPINA12* from *SERPINB7* and may contribute to the more pronounced inflammatory phenotype observed in SERPINA12-associated PPK (18).

One of the most consistent observations across all computational analyses was the prominent enrichment of extracellular matrix-related pathways following both *SERPINA12* and *SERPINB7* deletion. GO enrichment analysis identified NABA Core Matrisome, NABA Matrisome Associated, and extracellular matrix organization among the most significantly enriched biological processes. Similarly, KEGG analysis indicated enrichment of ECM–receptor interaction and focal adhesion pathways. Furthermore, extracellular matrix-related genes such as *COL1A1*, *COL1A2*, *FN1*, *SPARC*, *COL4A2*, and *MMP2* were among the most significantly perturbed genes following virtual knockout. Collectively, these findings suggest that both *SERPINA12* and *SERPINB7* may contribute to extracellular matrix homeostasis.

The extracellular matrix has traditionally been viewed as a structural scaffold supporting epidermal architecture (26–28). However, increasing evidence indicates that the extracellular matrix functions as a dynamic signaling platform that regulates keratinocyte proliferation, differentiation, migration, and tissue repair (29). Perturbations of extracellular matrix composition can disrupt communication between epidermal and dermal compartments and ultimately result in abnormal keratinization (30). Therefore, the consistent enrichment of extracellular matrix-associated pathways observed in both virtual knockout models provides a plausible molecular explanation for the highly similar clinical manifestations observed in SERPINA12-associated PPK and SERPINB7-associated NPPK.

Interestingly, correlation analysis imply that *SERPINB7* exhibited a particularly strong association with extracellular matrix-related gene signatures. This observation is consistent with previous studies suggesting that *SERPINB7* functions primarily in maintaining epidermal integrity and protecting tissues from excessive protease-mediated damage (15, 16). The predominance of extracellular matrix and structural remodeling pathways in the *SERPINB7* knockout model further supports the concept that disruption of tissue architecture represents a central pathogenic mechanism in NPPK (31, 32).

Despite the substantial overlap between the two virtual knockout models, our analyses also revealed important functional differences. Although global enrichment profiles were broadly similar, analysis of the most significantly perturbed genes identified a distinct immune-related signature associated with *SERPINA12* deficiency. GO enrichment analyses demonstrated significant enrichment of leukocyte activation, positive regulation of T-cell proliferation, immune effector regulation, and interleukin signaling. Consistently, KEGG pathway analysis revealed enrichment of Toll-like receptor signaling and complement-associated pathways following *SERPINA12* deletion. These observations suggest that *SERPINA12* may possess biological functions extending beyond extracellular matrix regulation and may additionally participate in the control of inflammatory responses.

The identification of Toll-like receptor signaling is particularly noteworthy. Toll-like receptors serve as key sensors of tissue injury and microbial products and play central roles in orchestrating innate immune responses within the skin (33). Activation of Toll-like receptor pathways promotes the production of pro-inflammatory cytokines and chemokines, thereby amplifying local inflammatory responses (34). Similarly, complement activation has emerged as an important contributor to inflammatory skin diseases through its ability to recruit immune cells and enhance cytokine production (35). The enrichment of both Toll-like receptor and complement pathways following *SERPINA12* deletion therefore suggests that loss of *SERPINA12* function may facilitate inflammatory activation within the epidermal microenvironment.

An important strength of the present study is the inclusion of experimental validation. To verify the computational predictions, *SERPINA12* was overexpressed in TNF-α-stimulated HaCaT keratinocytes. Remarkably, *SERPINA12* overexpression significantly reduced the expression of several inflammatory mediators, including TNF-α, IL-8, and IL-1β. Among these cytokines, IL-8 exhibited the most pronounced reduction, decreasing by more than 50% following *SERPINA12* overexpression. In contrast, IL-6 expression was not significantly altered. These findings provide direct experimental evidence that *SERPINA12* exerts anti-inflammatory effects in keratinocytes.

Interestingly, correlation analyses revealed only modest associations between *SERPINA12* expression and inflammation-related gene signatures. At first glance, this finding may appear inconsistent with the enrichment of multiple immune-related pathways and the experimental observations. However, correlation analyses primarily reflect steady-state transcriptional relationships, whereas virtual knockout modeling and gain-of-function experiments assess functional consequences following perturbation of gene activity. Therefore, a gene may exert substantial regulatory effects despite showing relatively weak co-expression with downstream targets. The consistency between virtual knockout predictions and experimental validation suggests that *SERPINA12* likely acts as an upstream regulatory molecule whose biological effects become evident when its expression is altered.

The biological significance of *SERPINA12* in inflammatory regulation is also supported by previous studies (36). *SERPINA12*, also known as vaspin, was originally identified as an adipokine with insulin-sensitizing properties. A previous case–control study conducted in a Turkish population demonstrated a significant association between the vaspin rs2236242 polymorphism and psoriasis susceptibility. Specifically, individuals carrying the TA genotype exhibited a 2.38-fold increased risk of developing psoriasis compared with those carrying the TT genotype. These findings suggest that genetic variation in the vaspin gene may contribute to psoriasis pathogenesis and support its potential role as a diagnostic biomarker (37). Subsequent investigations have demonstrated anti-inflammatory effects of vaspin in multiple tissues and disease models. *SERPINA12* has been reported to suppress inflammatory cytokine production, attenuate NF-κB signaling, and protect against tissue injury in various inflammatory conditions. Although its role in skin biology remains poorly understood, our findings suggest that similar anti-inflammatory mechanisms may operate within keratinocytes and contribute to epidermal homeostasis.

Taken together, our data support a model in which *SERPINA12* and *SERPINB7* share a common extracellular matrix-associated pathogenic framework but diverge in their influence on inflammatory signaling. Extracellular matrix dysregulation likely explains the substantial clinical overlap between SERPINA12-associated PPK and SERPINB7-associated NPPK. In contrast, the additional anti-inflammatory function of *SERPINA12* may account for the more pronounced inflammatory manifestations observed in patients carrying *SERPINA12* variants. This model provides a molecular explanation for the coexistence of phenotypic similarity and disease-specific differences.

Several limitations should be acknowledged. First, we acknowledge that GSE130973 was generated from healthy skin rather than palmoplantar keratoderma lesions. Owing to the lack of publicly available single-cell RNA sequencing datasets from SERPINA12- or SERPINB7-associated palmoplantar keratoderma, GSE130973 was selected to investigate the predicted regulatory consequences of gene deficiency. Therefore, our findings should be interpreted as hypothesis-generating predictions requiring future validation in disease-specific samples. Second, although the present study combined computational analyses with experimental validation, the molecular mechanisms linking *SERPINA12* to inflammatory signaling remain incompletely understood. Our overexpression experiments were primarily designed to provide preliminary functional evidence supporting the computational predictions rather than to comprehensively model PPK pathology. Future studies should investigate keratinocyte differentiation, epidermal barrier integrity, keratinization, and extracellular matrix remodeling to further establish the mechanistic role of SERPINA12 in PPK. Third, the anti-inflammatory effects of *SERPINA12* were evaluated in an overexpression model, which does not directly recapitulate the loss-of-function state observed in disease, and future studies employing loss-of-function approaches will be required to establish causality more definitively. Overexpression was selected because previous (38) studies have reported that SERPINA12 is absent or expressed at extremely low levels in HaCaT cells. Consistent with these reports, we were unable to detect endogenous SERPINA12 expression in HaCaT cells by qPCR, Western blotting, or RNA sequencing, precluding meaningful knockdown experiments in this cell line. Therefore, ectopic overexpression was adopted as the most feasible strategy to investigate the biological function of SERPINA12. Finally, the downstream signaling pathways through which *SERPINA12* regulates cytokine production remain to be elucidated.

Although the present study focused on hereditary palmoplantar keratoderma, the anti-inflammatory effect of *SERPINA12* observed in TNF-α-stimulated HaCaT keratinocytes may also have implications for psoriasis-like epidermal inflammation. Previous studies have reported altered circulating levels of *SERPINA12*, also known as vaspin, in patients with psoriasis, suggesting a potential relationship between *SERPINA12* and systemic inflammatory status in psoriatic disease (39–41). Importantly, the overexpression experiments were not intended to mimic the disease-associated loss-of-function state, but rather to provide complementary functional evidence supporting the predictions generated by the virtual knockout analysis. Future studies using psoriasis-relevant keratinocyte models, patient-derived skin samples, and mechanistic analyses of downstream inflammatory pathways will be needed to clarify whether *SERPINA12* contributes to the regulation of psoriatic inflammation.

In conclusion, this study provides the first comparative molecular characterization of *SERPINA12* and *SERPINB7* deficiency. Our findings indicate that both genes may participate in extracellular matrix homeostasis, supporting a shared pathogenic mechanism underlying their similar keratoderma phenotypes. However, *SERPINA12* additionally may participate in the inhibition of inflammatory signaling pathways and cytokine expression in keratinocytes. These observations provide a plausible molecular explanation for the enhanced inflammatory manifestations associated with SERPINA12-related PPK and expand our understanding of the diverse biological functions of SERPIN family members in skin homeostasis and disease.

## Data Availability

The datasets presented in this study can be found in online repositories. The names of the repository/repositories and accession number(s) can be found in the article/Supplementary material.
