# Supplementary material for: Integrated bioinformatics analysis reveals convergent molecular signatures associated with SERPINB7 and SERPINA12 deficiency
Source: Front Med (Lausanne). 2026 Aug 20;13:1920631. doi: 10.3389/fmed.2026.1920631 (PMC13537938; doi:10.3389/fmed.2026.1920631)
Supplement: Supplementary file 1 [file Table_1.docx]

**Figure S1. Workflow of the virtual knockout analysis using the scTenifoldKnk algorithm.**

The workflow illustrates the computational pipeline used in this study. The processed single-cell RNA sequencing dataset (GSE130973) was used as the input for virtual knockout analysis. Gene regulatory network perturbations following **SERPINA12** or **SERPINB7** virtual knockout were predicted using the **scTenifoldKnk** algorithm. The resulting predicted regulatory perturbation genes were subsequently subjected to Gene Ontology (GO), Kyoto Encyclopedia of Genes and Genomes (KEGG), Reactome, and Molecular Signatures Database (MSigDB) enrichment analyses to identify potentially affected biological processes and signaling pathways. The computational workflow was designed to generate hypotheses regarding the downstream molecular mechanisms associated with SERPINA12 and SERPINB7 deficiency.
